# First Molecular Detection of Torque Teno Canis Virus in Apparently Healthy Dogs in Southern Italy

**DOI:** 10.1002/vms3.70698

**Published:** 2025-12-16

**Authors:** Gianmarco Ferrara, Ugo Pagnini, Francesco Origgi, Serena Montagnaro

**Affiliations:** ^1^ Department of Veterinary Sciences University of Messina, Polo Universitario dell'Annunziata Messina Italy; ^2^ Department of Veterinary Medicine and Animal Productions University of Naples Federico II Naples Italy

**Keywords:** anelloviridae, canine virus, Torque teno canis virus, TTV

## Abstract

**Background:**

Torque teno viruses are viruses with unclear pathogenic potential, as conflicting evidence has classified this family of viruses as harmless and opprtunistic. Dogs also have their own specific Torque teno virus, called Torque teno canis virus (TTCaV), which has been reported in several countries.

**Objectives:**

This study aimed to identify TTCaV in the feces of apparently healthy dogs and to evaluate risk factors correlated to higher prevalences.

**Methods:**

Faecal samples were collected from 171 dogs from the Campania region, Italy. DNA was extracted from each sample and used as a template in a nested end‐point PCR, and some positive samples were sequenced by the Sanger method. Univariate analysis was performed to assess the correlation between molecular detection of TTCaV and variables included in the study.

**Results:**

A total of 18 out of 171 animals (10.5%) were PCR‐positive. No individual factor (sex, age, etc.) was associated with higher prevalence, while significant differences were observed in mixed‐breed (16.5%) and kennel dogs (17.7%). Although the identification of TTCaV DNA was more frequent in dogs with an altered faecal score (score > 2 on a 1–7 scale), we did not find higher prevalence in CPV‐2‐positive animals (even if based on only 11 CPV‐2‐positive samples). A total of six amplicons were sequenced, obtaining two different isolates that, once deposited in international databases and compared with those reported in other studies, showed a homology with other strains identified worldwide.

**Conclusions:**

Although questions concerning the clinical relevance of TTVs still remain unanswered, our study documented the presence of this virus in the dog population in southern Italy. Moreover, our work provided phylogenetic data and useful information to better characterize the epidemiological picture of this virus in Italy and in Europe.

## Graphical abstract

•Torque teno canis virus has been detected in dogs from southern Italy.

•Detection of TTCaV DNA was more frequent in dogs with an altered faecal score, mixed‐breed and belonging to kennels.

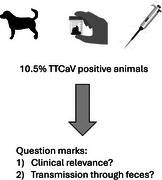

## 1 Introduction

The *Anelloviridae* family includes small non‐enveloped viruses classified into two genera: genus *Alphatorquevirus* (TTVs) and genus *Betatorquevirus* (TTMVs). The first Torque teno virus (TTV) ever described was identified in a patient with post‐transfusion hepatitis in 1997 (Focosi et al. 2016). The newly discovered virus was first proposed in the *Circoviridae* family (subsequently counted in the *Anelloviridae* family) due to its circular and single‐stranded DNA (2.1–3.8 kb size) (Focosi et al. 2016; Webb et al. 2020).

Since its discovery, TTV has been identified in a high number of people and has been detected in several specimens (as serum, saliva, feces, semen, kidney, liver and urine) (Hino and Miyata 2007). Despite hundreds of worldwide reports, the biological nature of TTV is yet to be characterized secondary to the not yet established clinical relevance and exact molecular mechanisms of viral replication (Focosi et al. 2016; Spezia et al. 2023). TTV has been proposed to be associated in humans (without evidence of direct involvement) with several pathologies, such as hepatic and pulmonary diseases, haematologic disorders, idiopathic inflammatory myopathy and systemic lupus erythematosus (Hino and Miyata 2007; Sabbaghian et al. 2024; Webb et al. 2020).

Over the years, thanks to the advent of the latest generation of molecular methods, TTV‐like viruses have been described in numerous animal species (chimpanzees, gorillas, tupaias, chickens, pigs, sheep, cattle, sea lions, cats, rodents and dogs) (Cavalcante et al. 2023; Hrazdilová et al. 2016; Okamoto 2009; Okamoto et al. 2002; Y. Wu et al. 2018). Specific TTVs have even been identified in ticks, in some cases sharing discrete homologies with the TTVs detected in their hosts (Waits et al. 2018). Given that the genomic organization and transcriptional profiles of TTVs infecting nonhuman primates and other mammalian species are comparable to those of human TTVs, co‐evolution between TTVs and their hosts has been hypothesized. Although they were hypothesized as co‐infecting viruses, only Swine Torque teno virus (TTSuV) was established to exacerbate porcine circovirus (PCV‐2) infection in pigs. In fact, TTSuV contributes to the development of nephropathy and dermatitis during experimental infection (Kekarainen and Segalés 2012; Webb et al. 2020). Nowadays, TTSuV is the only TTV whose immunization employing by a combining DNA and protein vaccine, has been demonstrated to effectively suppress TTSuV viremia (Jiménez‐Melsió et al. 2015).

Torque teno canis virus (TTCaV), a species belonging to the Thetatorquevirus genus, was first described in Japan in 2002 (Belák et al. 2013; Okamoto et al. 2002). The TTCaV is a small and non‐enveloped DNA virus that has been reported to cause infections in dogs. The genome (approximately 2.8 kb in length) shows the classical TTV structure (sharing less than 50% of sequence homology with human TTV) with three major open reading frames (ORF1, ORF2 and ORF3) and a short stretch of untranslated region (UTR) with high GC content (Wang et al. 2023). ORF1 encodes the nucleocapsid, which is a structural protein, whereas ORF2 encodes a nonstructural protein required for viral replication (Belák et al. 2013). The ORF3 protein works similarly to phosphorylated viral proteins (Wang et al. 2023). Reports describing the presence of this virus in various dog specimens are limited to Asian countries (China, Japan, Korea, etc.), while there is no evidence that these viruses are also widespread in Europe (Kim et al. 2022; Lan et al. 2011; Sun et al. 2017; Wang et al. 2023).

The present study aimed to identify the presence of TTCaV in faecal samples obtained from clinically healthy dogs from the Campania region, southern Italy. Although the prevalence appears to be higher in certain matrices (e.g., serum or blood), the choice of sample was faeces since it can represent a route of viral shedding and hence a possible candidate for transmission to other dogs (Lan et al. 2011; Turan et al. 2020; Kim et al. 2022). Since TTCaV's pathogenic role and its significance in co‐infection with other pathogens have yet to be studied, we also investigated the association of TTCaV with faecal score and canine parvovirus 2 (CPV‐2), a primary enteric pathogen in dogs.

## 2 Materials and Methods

### 2.1 Sample Collection and DNA Extraction

Fresh faecal samples were collected from 171 dogs belonging to the Campania region, Southern Italy (40°49′34″N 14°15′23″E). The sample size was calculated using Thrusfield's approach for a theoretically ‘infinite’ population, which contained the following information: estimated TTV prevalence (10%), 95% confidence interval (CI) and desired absolute precision (5%). A total of 43 hunting dogs, 79 stray dogs and 49 pet dogs were sampled. A questionnaire was employed to acquire information about each sampled animal (sex, breed, size, location, age, origin, lifestyle and attitude) (Ferrara et al. 2024, 2025). All animals included in this study were clinically healthy (including those that tested positive for CPV‐2 in subsequent molecular analyses) (Ferrara et al. 2025). Furthermore, a score was assigned to each sample based on its physical features as described in previous studies (ranging from 1 = *solid* to 7 = *watery*) (Lappin et al. 2022). Following collection, each specimen was immediately processed into 10% (w/v) suspensions in phosphate‐buffered saline 1X (PBS, pH 7.2‐7.4) and centrifuged for 10 min at 1500 g. Viral DNA was extracted from the supernatants using QIAamp Fast DNA Stool Mini Kit and following the manufacturer's instructions. Each DNA sample was extracted and then kept at ‐80°C until it was processed.

The animal study protocol was approved by the Institutional Ethics Committee of the Department of Veterinary Medicine and Animal Production (Centro Servizi Veterinari), University of Naples, Federico II (PG/2022/0093420, 21st July 2022).

### 2.2 Molecular and Statistical Analysis

TTCaV was detected by amplification of a 385‐bp fragment using a nested polymerase chain reaction (PCR) protocol described in a previous study (Lan et al. 2011; Sun et al. 2017). The first amplification step included the following steps: an initial denaturation at 94°C for 5 min, followed by 35 cycles consisting of 94°C for 40 s (denaturation), 50°C for 40 s (annealing) and 72°C for 90 s (extension). A final extension for 10 min at 72°C followed (primers and amplicon size were listed in Table 2) (Lan et al. 2011; Sun et al. 2017). The second‐round PCR included the same thermal conditions but with different primers, and as template, the amplified product from the first step (listed in Table 1). All the amplification steps were performed using the Taq DNA Polymerase kit (QIAGEN) following the manufacturer's instructions. PCR products were resolved in electrophoresis gels (containing 1.5% agarose) in order to verify the amplicon size. The same samples were also evaluated for the presence of canine parvovirus‐2 (CPV‐2) using a real‐time PCR protocol described in the literature (primers are listed in Table 1) (Decaro et al. 2005). The real‐time reaction was performed with the iTaq Universal Probes Supermix kit (Biorad) and read with a CFX96 Real‐Time PCR Detection System (Bio‐Rad). Chi‐square statistics were used to analyse the relationship between the dependent (PCR outcome) and independent variables (as obtained through the questionnaire). The independent variables were age (≤ 2 years considered young, > 2 and ≤ 6 years considered adults, > 6 years considered old), sex (male or female), location (Avellino, Benevento, Salerno, Caserta, Napoli), attitude (hunting and non‐hunting), breed (mix or pure breed), origin (private or stray), faecal score (normal = ≤ 2 and not normal > 2) and CPV‐2 status (positive or negative). To eliminate bias, multiple laboratory personnel performed faecal score evaluation processes and molecular diagnostic techniques in a double‐blind approach. Statistical analysis was performed using MedCalc Statistical Software version 16.4.3 (MedCalc Software, Ostend, Belgium) and JMP version 14.1.0 (SAS Institute Inc.), considering significant *p* values lower than 0.05.

**TABLE 1:** Primers and probes used in this study for the end‐point amplification of the ORF1 sequence (TTCaV) and real‐time PCR of the VP‐2 sequence (CPV‐2).

	Forward	Reverse	Size (bp)
TTV‐First step	AACATCACAAATACCCATTAACATTCCC	TGCTGTCGCTGTCTTCGCTCAC	453
TTV‐Second step	CCAAGGGACCAGCACCCACATT	CTGTCGCTGTCTTCGCTCACCC	385
CPV‐2^a^	AAACAGGAATTAACTATACTAATATATTTA	AAATTTGACCATTTGGATAAACT	125

^a^ For the real‐time amplification of the VP‐2, the following probe was also used FAM‐TGGTCCTTTAACTGCATTAAATAATGTACC.

Based on DNA integrity and purity, only a total of six positive samples were selected, including both the first and second steps of the nested PCR, which were processed using a commercial kit (QIAquick PCR Purification Kit, Qiagen) and Sanger sequenced using the primers described previously. The sequences obtained in this study were analysed using MEGA software (MEGA 10), aligned and compared to TTV sequences described in other studies and available through international databases (GenBank, NCBI). A maximum likelihood phylogenetic tree of the partial ORF1 was then obtained using the Bootstrap method (number of Bootstrap replications equal to 150).

## 3 Results

A total of 18 samples were positive out of the 171 tested, resulting in a molecular prevalence of 10.5% (95% CI 5.9–15.1, Table 2). TTCaV was identified homogeneously in all provinces (without significant differences), demonstrating the distribution of the virus in the entire study area. Among the risk factors analysed, several were statistically associated with higher prevalence (Table 2). Dogs from kennels and mixed breeds were more likely to test positive. Although positive results were more frequent in stray and outdoor animals, it was not possible to establish a statistically significant correlation with these risk factors. Dogs with an altered faecal score (greater than 2), however, presented higher molecular prevalence (16%, 95% CI 8.1–24). Furthermore, we did not find any significant difference based on sex, age or CPV‐2 positivity (*C*_t_ ranging from 21 to 33 using a cut‐off of 35). In particular, a dog was found to be co‐infected with TTCaV and CPV‐2 (coinfection of 0.6% of the total and 5.5% of TTCaV‐positive animals). Moreover, *C*_t_ values of CPV‐2 positive samples did not correlate with TTCaV positivity

**TABLE 2:** Univariate analysis of potential risk factors for Torque teno canis virus (TTCaV) detection.

	TTCaV					
Factor	*n*	Positive	%	95% CI	*χ*2	*p*
Total	171	18	10.5	5.9–15.1		
						
Province					2.7	0.6
Avellino	32	4	12.5	1–24		
Benevento	20	2	10	0–23.1		
Salerno	46	4	8.7	0.5–16.8		
Caserta	39	4	10.2	0.7–19.8		
Napoli	18	4	22.2	3–41.4		
Sex					0.4	0.5
Male	101	12	11.9	5.6–18.2		
Female	70	6	8.6	2–15.1		
Age					2.8	0.2
Young	34	2	5.9	0–13.8		
Adult	82	12	14.6	7–22.3		
Old	55	4	7.3	0.7–14.1		
Attitude					8.4	**0.01** ^a^
Hunting	43	1	2.3	0–6.8		
Kennel	79	14	17.7	9.3–26.1		
Pet	49	3	6.1	0–12.8		
Breed					6.3	**0.01** ^a^
Mix	85	14	16.5	8.6–24.4		
Specific breed	86	4	4.6	0.2–9.1		
Origin					2.9	0.09
Stray	91	13	14.3	7.1–21.5		
Housed	80	5	6.2	0.9–11.5		
Faecal score					5	**0.03** ^a^
≤ 2	90	5	5.6	0.8–10.3		
> 2	81	13	16	8.1–24		
Life					0.77	0.38
In	43	3	7	0–14.6		
Outside	128	15	11.7	6.1–17.3		
CPV‐2 status					0.03	0.87
Positive	11	1	9.1	0–26.1		
Negative	160	17	10.6	5.8–15.4		

Abbreviations: CI, confidence intervals; CPV‐2, canine parvovirus type 2; *χ* ^2^, chi square.

^a^statistical significance.

**FIGURE 1:**
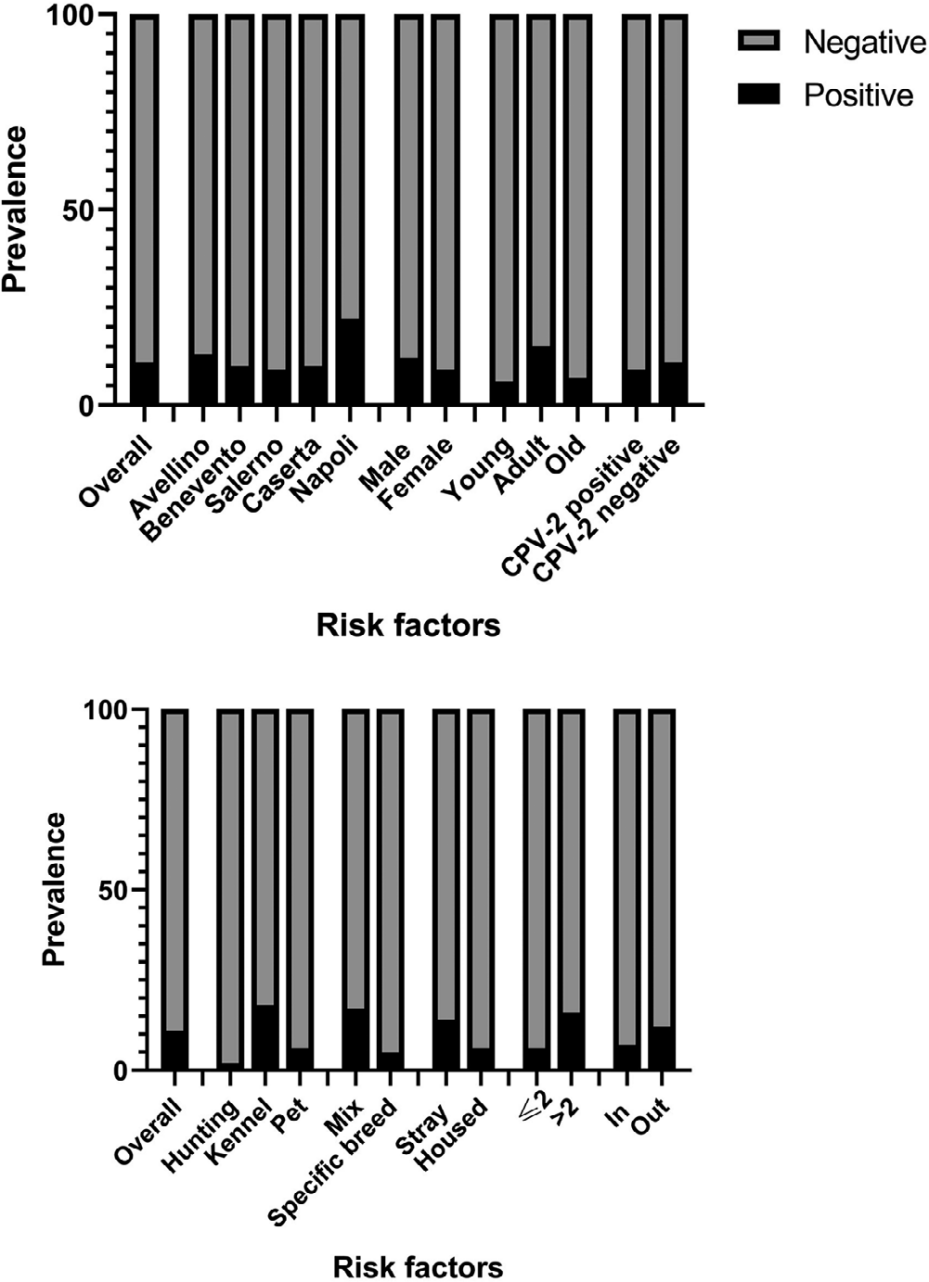
Bar graph represents the TTCaV prevalence in the study population. This bar graph shows the trend in the prevalence of TTCaV in different categories. Significant prevalence rates were found in the province of Naples, in shelter dogs with faecal scores greater than 2.

**FIGURE 2:**
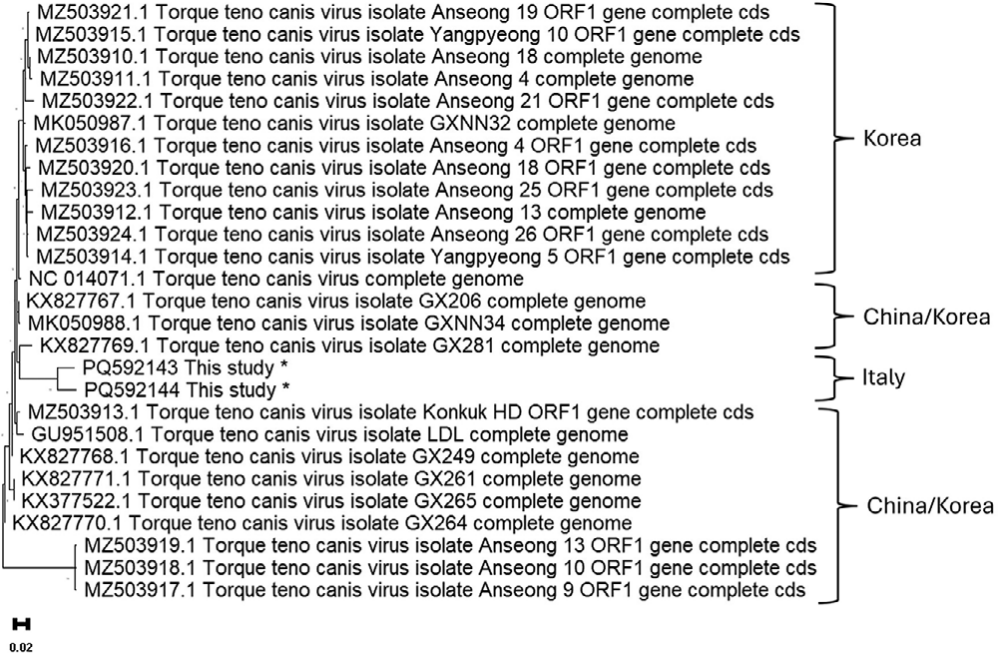
Phylogenetic analyses of Torque teno canis virus based on the full sequence of ORF1. A Phylogenetic tree obtained by aligning the partial ORF1 sequences of Torque teno canis virus sequenced in this study (indicated with “THIS STUDY”) and the 25 strains described in other studies. The country of origin and accession numbers (GenBank) are indicated.

The sequences obtained from six positive samples were aligned and compared with those deposited in international databases, obtaining high nucleotide similarity (up to 97% identity). They differed from each other by 15 base pairs in length and clustered separately. The phylogenetic tree obtained using the available sequences (belonging exclusively from Asia) showed the novel sequences obtained clustering between the South Korean and Chinese strains (Figures 1 and 2).

## 4 Discussion

This study described for the first time the detection of TTCaV in dogs in Europe and specifically in Italy. Because this is the first report of TTCaV in Italy, it would be important to underline whether the virus was present in the past or was introduced lately (for example, through animal movements or domestic‐wildlife interactions). The prevalence described by our study (10.5%) was similar to that reported in other studies carried out in Asia (particularly in China and South Korea). These studies, in fact, reported prevalence of 7%, 13% and 16.5% in faecal samples from three different regions of China and 9.6% in South Korea (Kim et al. 2022; Lan et al. 2011; Sun et al. 2017; Wang et al. 2023). In this latest study, the prevalence of TTCaV in dog faeces also differed based on the region (ranging from 5% to 24%). Moreover, higher prevalence has been described in serum samples from Japan (38%) and faecal samples from Turkey (32.8%) (Okamoto et al. 2002; Turan et al. 2020). A study carried out in Brazil reported the presence of the tatorquevirus in the pooled serum of dogs using a metagenomic approach (Weber et al. 2018). The observed prevalence must be considered underestimated, as a greater prevalence would be predicted when utilizing blood/serum as a matrix, given that viremia is more constant and detectable. The use of solely faecal samples for TTCaV detection may not be meant as a significant constraint considering the accessibility of faecal samples and the possibility of investigating excretion. However, reported prevalence varies from the method applied (PCR or metagenomic approach), type of sample used (faeces, faecal swab or serum) and the inclusion of clinically healthy or diseased animals (as well as any epidemiological differences existing between the various countries). Although TTCaV has not been reported in Europe, other TTV‐like viruses have been described in other animal species (pigs, horses, wildlife, etc.) (Fisher et al. 2023; Kekarainen and Segalés 2012). In particular, numerous studies have highlighted the spread of TTSuV in both pigs and wild boars in Europe, and a pathogenic role in exacerbating clinical signs following PCV‐2 infection (Singh and Ramamoorthy 2018; Webb et al. 2020; Z. Wu et al. 2011; Manzin et al. 2015; Martínez et al. 2006). Researchers are attempting to identify a pathogenic function for TTCaV in dogs as well as a relationship with coinfection. A study carried out in China reported for the first time a coinfection of TTCaV and canine astrovirus (CaAstV) in a small percentage of samples (Zhang et al. 2020). However, a role has not yet been attributed to TTCaV (nor does its frequency appear to be more prevalent in dogs co‐infected with other viruses such as CPV‐2). CPV‐2 and TTCaV coinfection was previously documented in an investigation carried out in China (although without carrying out a statistical analysis) (Sun et al. 2017). It would be interesting to investigate if, on the other side, TTCaV detection is more common in CPV‐2‐positive (clinically ill) animals. The results of our study, however, highlighted a greater frequency of detection of the virus in animals that presented loose stools (with a faecal score greater than 2), raising suspicions about the potential pathogenic role of this virus alone or in association with other pathogens. This finding requires more investigation to rule out the possibility that TTCaV detection is simpler in animals with an altered faecal score due to higher elimination, which is independent of the potential pathogenic role.

Based on the analysis of the sequences obtained, we hypothesized the presence of two different TTCaV clusters circulating in the Campania region (which differed from each other by 15 pairs of bases). No differences were observed in the types of dogs infected by the two clusters. Furthermore, these strains displayed high degrees of homology with those described in Asia, demonstrating that the genome of these viruses is rather conserved worldwide (Kim et al. 2022; Lan et al. 2011; Sun et al. 2017; Wang et al. 2023). Interestingly, a recent study showed the homology of the various isolates acquired from felid samples (pumas, ocelots, bobcats, caracals, cats and lynxes). Also in that case, ORF1 protein sequence similarity analysis revealed two separate clusters included in the felid‐derived anellovirus sequences, which mirrors the pattern reported in pigs (Kraberger et al. 2021).

Similarly, the transmission route of TTCaV is not yet clear as well, whereas for the human counterpart, both the inhalation and faecal‐oral routes have been demonstrated (Spezia et al. 2023). Our study was the first to evaluate possible risk factors for TTCaV positivity in dogs. The data obtained identified greater risks in kennel and mixed‐breed dogs. This data could indicate that overcrowded conditions would facilitate the transmission of this virus, while individual factors (age, sex, etc.) did not correlate to a higher prevalence (Petruccelli et al. 2021). A study carried out in Korea also provided supporting arguments for a higher prevalence in the Anseong region (although it did not statistically evaluate the results) by asserting that the samples collected were mainly shelter dogs (Kim et al. 2022).

Given that the pathogenic role has not been defined, the prevalent literature has suggested anelloviruses as benign commensals or at most opportunistic agents; therefore, the presence of this virus has been used as a potential marker for different human disease conditions. It is tempting to think of potential similar applications, also in veterinary medicine. For example, based on the continuous virus presence in the blood system (TTVs were tolerated by their hosts), a study employed the quantification of TTV in body fluids as a marker of immunosuppression (Timmerman et al. 2024). Another study demonstrated that the TTV load increases during complications after allogeneic haematopoietic cell transplantation (allo‐HCT) (Srour et al. 2024). Likewise, a further study employed TTV as a marker of anthropic pollution (Focosi et al. 2016). Another study evaluated the viral load of TTV in wastewater and drinking water samples as an indicator of faecal contamination, identifying a prevalence of 38%–100% of wastewaters, 25% of waters influenced by waste discharges, and 5% of waters used as drinking water sources (Charest et al. 2015). Although not relevant to the findings of this study, this evidence suggests that future studies should establish the potential these viruses have for veterinary medicine.

Another important question mark is the possible transmission of these viruses to other species. Combined high serological and molecular prevalence rate of TTSuV was reported in samples belonging to horses, cattle, dogs, sheep and elk (Singh and Ramamoorthy 2018). Heterologous infections between humans and pigs were reported to occur between their respective TTVs (Ssemadaali et al. 2016). Similarly, detection of heterologous anelloviruses was observed in the Campania region, where TTV was reported in buffalo milk and dairy products (Roperto et al. 2009) and in wildlife (chamois and deer), which tested positive for TTSuV in liver samples (Righi et al. 2022). Although these data suggest the wide host range of species‐specific TTVs, there is still no evidence of the transmissibility of TTCaV to other species, as well as its pathogenic role. Further studies are required to understand the real consequences of TTCaV‐1 infection, its possible role as an opportunist and its potential transmission to other species.

## 5 Conclusion

In the present study, we have detected TTCaV nucleic acids in the faeces of clinically healthy dogs, identifying some risk factors associated with a higher risk of being shedders, such as origin (kennel), breed (crossbreed) and faecal score. Although this study showed some limitations (as the absence of viral quantification), it represents the first description in Europe of this virus in the canine population and contributes to questioning the actual pathogenicity of TTCaV.

## Author Contributions

**Gianmarco Ferrara**: conceptualization; investigation; writing – original draft; methodology; writing – review and editing; software; data curation. **Ugo Pagnini**: conceptualization; investigation; validation; visualization; writing – review and editing; supervision. **Francesco Origgi**: supervision; data curation; resources; formal analysis; writing – review and editing. **Serena Montagnaro**: resources; supervision; formal analysis; writing – review and editing.

## Funding

The authors have nothing to report.

## Ethics Statement

The animal study protocol was approved by the Institutional Ethics Committee of Department of Veterinary Medicine and Animal production (Centro Servizi Veterinari), University of Naples, Federico II (PG/2022/0093420 20 July 2022).

## Conflicts of Interest

The authors declare no conflicts of interest.

## Data Availability

The sequences obtained in the current study are available at GenBank, accession numbers PQ592143 and PQ592144.
